# Application of mechanical cardiopulmonary resuscitation devices and their value in out-of-hospital cardiac arrest: A retrospective analysis of the German Resuscitation Registry

**DOI:** 10.1371/journal.pone.0208113

**Published:** 2019-01-02

**Authors:** Stephan Seewald, Manuel Obermaier, Rolf Lefering, Andreas Bohn, Michael Georgieff, Claus-Martin Muth, Jan-Thorsten Gräsner, Siobhán Masterson, Jens Scholz, Jan Wnent

**Affiliations:** 1 Institute for Emergency Medicine and Department of Anaesthesiology and Intensive Care Medicine, Schleswig-Holstein University Hospital, Campus Kiel, Kiel, Germany; 2 Department of Anaesthesiology, Heidelberg University Hospital, Heidelberg, Germany; 3 Institute for Research in Operative Medicine, Faculty of Medicine, University of Witten/Herdecke, Cologne, Germany; 4 City of Muenster, Fire Department, Muenster, Germany; 5 Department of Anaesthesiology, Ulm University Hospital, Ulm, Germany; 6 Section of Emergency Medicine, Department of Anaesthesiology, Ulm University Hospital, Ulm, Germany; 7 Discipline of General Practice, National University of Ireland Galway, Newcastle, Galway, Ireland; 8 Schleswig-Holstein University Hospital, Kiel, Germany; Medizinische Hochschule Hannover, GERMANY

## Abstract

**Background:**

Cardiac arrest is an event with a limited prognosis which has not substantially changed since the first description of cardiopulmonary resuscitation (CPR) in 1960. A promising new treatment approach may be mechanical CPR devices (mechanical CPR).

**Methods:**

In a retrospective analysis of the German Resuscitation Registry between 2007–2014, we examined the outcome after using mechanical CPR on return of spontaneous circulation (ROSC) in adults with out-of-hospital cardiac arrest (OHCA). We compared mechanical CPR to manual CPR. According to preclinical risk factors, we calculated the predicted ROSC-after-cardiac-arrest (RACA) score for each group and compared it to the rate of ROSC observed. Using multivariate analysis, we adjusted the influence of the devices’ application on ROSC for epidemiological factors and therapeutic measures.

**Results:**

We included 19,609 patients in the study. ROSC was achieved in 51.5% of the mechanical CPR group (95%-CI 48.2–54.8%, ROSC expected 42.5%) and in 41.2% in the manual CPR group (95%-CI 40.4–41.9%, ROSC expected 39.2%). After multivariate adjustment, mechanical CPR was found to be an independent predictor of ROSC (OR 1.77; 95%-CI 1.48–2.12). Duration of CPR is a key determinant for achieving ROSC.

**Conclusions:**

Mechanical CPR was associated with an increased rate of ROSC and when adjusted for risk factors appeared advantageous over manual CPR. Mechanical CPR devices may increase survival and should be considered in particular circumstances according to a physicians’ decision, especially during prolonged resuscitation.

## Introduction

The quality of Cardiopulmonary Resuscitation (CPR) is important for resuscitation success. With manual CPR, increasing fatigue of the rescuers and frequent interruptions of compressions have been reported.[1–3] Fatigue and interruptions decrease blood flow required for adequate myocardial and brain perfusion, which is crucial for good neurological outcome.[4, 5] High fraction chest compressions have been shown to lead to a higher rate of survival, compared with insufficiently performed chest compressions.[6–9]

The issues encountered with manual CPR led to the development of mechanical CPR systems. These devices perform chest compressions mechanically and automatically through inflatable vests, mechanical pistons, or load distributing bands. In Table 1 we describe the devices applied by emergency medical services (EMS) participating in the German Resuscitation Registry during our study period.

**Table 1:** Mechanical CPR devices applied by German Resuscitation Registry participants during our study period.

Device	AutoPulse	LUCAS
manufacturer	Zoll Medical, Chelmsford, USA (prior: Revivant)	Physio-Control, Redmond, USA and Jolife, Lund, Sweden
technique	load distributing band	mechanical piston
models and precursors	“AutoPulse” (different versions): electric driven “vest CPR” (discontinued): inflatable vest, pneumatic, circular pressure	“LUCAS 2”: electric driven “LUCAS”: gas-driven

Manufacturer information.

Applied studies have shown that using mechanical CPR devices, higher arterial carbon dioxide levels (P_a_CO_2_) can be achieved, as well as better haemodynamics, thereby leading to improved coronary and cerebral perfusion.[10] Furthermore it has been stated that coronary and brain perfusion is the leading determinant of survival following resuscitation. Based on the premise that mechanical CPR provides a sustained quality of chest compressions, better outcomes might be expected from mechanical CPR devices. Results from studies investigating the effect of mechanical CPR however, are mixed. Improved short-term neurological outcome, return of spontaneous circulation (ROSC), and short-term survival has been shown in some studies using mechanical CPR.[10, 11] Other investigations however, did show an association between the use of mechanical CPR with lower neurologically favourable survival after out-of-hospital cardiac arrest.[12, 13] Some authors have reported severe complications when mechanical CPR was used, including more frequent and traumatic injury. More interruptions caused by the application of mechanical CPR devices were observed, as well as worse compression quality, haemodynamic parameters, oxygen metabolism, and outcome.[7, 10, 14, 15]

In current guidelines, mechanical CPR devices were introduced as potential advanced life support (ALS) adjuvant. While the advantages and disadvantages mentioned above are discussed, no recommendations in favour of or against the application of these devices are given.[16] Therefore we conducted this study to work out the advantage or disadvantage in a register based analysis.

## Material and methods

The aim of this study was to analyse a large CPR database, the German Resuscitation Registry, to evaluate potential benefits of mechanical CPR devices over manual CPR in adult cardiac arrest victims. The primary endpoint considered is ROSC.

We conducted a retrospective analysis of the German Resuscitation Registry database. The registry is operated by the German Society of Anaesthesiology and Intensive Care Medicine and was founded in 2007. The participating centers pay furthermore an annual fee. The registry includes fully anonymized data from out-of-hospital (OHCA), in-hospital cardiac arrest (IHCA), and in-hospital emergency treatment. Data is gathered on both the emergency and in-hospital care of each patient. During the study period (1^st^ January 2007 to 31^st^ December 2014), 114 out-of hospital and 86 in-hospital sites provided data to the registry on a voluntary basis. A total of 42,141 resuscitations were documented: 6,548 in the hospital setting and 35,593 out-of-hospital. Protocol forms are available on the Internet (www.reanimationsregister.de).[17]

### Setting

The German EMS is a two-tiered, paramedic and emergency physician based system. Paramedic-staffed ambulances (mobile intensive care units) are dispatched for basic aid and patient transportation. If necessary, medic vehicles carry emergency physicians (mainly anaesthetists, surgeons, and internists after completing additional training for emergency medicine) to the incident location.

### Mechanical CPR devices

During the study period, the use of two devices, the LUCAS and AutoPulse, was documented in the registry (Table 1).

### Design

The documented cases were divided into a manual CPR group, and a mechanical CPR group.

#### Enrolment

Between January 1^st^, 2007 and December 31^st^, 2014, a total of 35,593 OHCA were documented in the out-of-hospital setting.

We excluded 15,984 cases (44.9%). We excluded cases in which CPR was continued for less than five minutes, or duration of CPR was missing, as the outcome in this early period is not affected by the CPR mode. Children aged less than 18 years and patients of unknown age were also excluded as the devices are not approved for resuscitation on children. An active compression-decompression (ACD) is a hand-held suction device, to compress and actively decompress the chest after each compression. We also excluded cases where ACD CPR was used, because it constitutes a different technology. Cases due to trauma were excluded as application of mechanical CPR devices may be limited due in traumatic events. Cases where data on ROSC and/or CPR mode was missing were also excluded.

**Fig 1:**
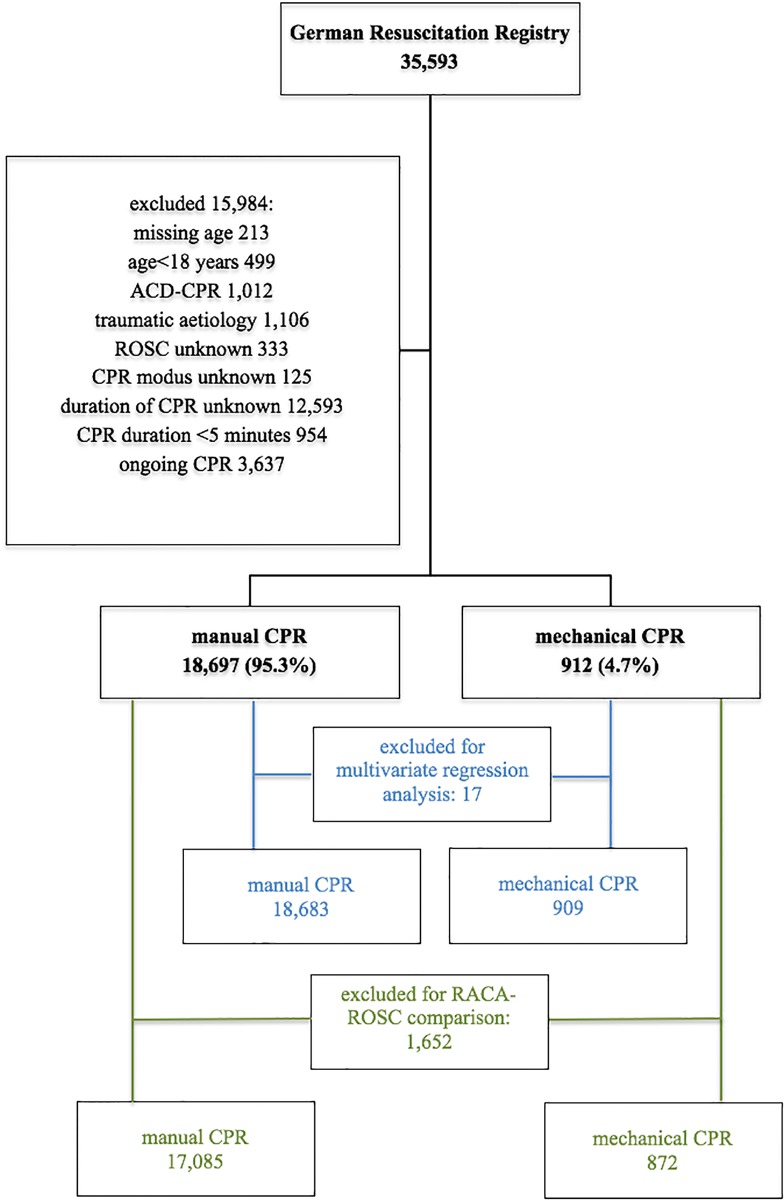
Enrollment. Enrolled: Preclinical setting from 01/01/2007 to 12/31/2014. Excluded cases: multiple entries are feasible. CPR = cardiopulmonary resuscitation; ACD = active compression and decompression; ROSC = return of spontaneous circulation.

The enrolment process and division into study groups is illustrated in Fig 1.

#### Definitions

We performed our definitions and statistics according to the Utstein Style.[18] By derogation from this, within the registry, return of spontaneous circulation (ROSC) is defined as palpable pulse maintained for at least 20 seconds.

We calculated the duration of CPR as the interval between initiation of CPR and the occurrence of first ROSC, or the termination of the resuscitation attempt. When the initiation time of CPR was unknown and bystander CPR had been performed, we assumed the time of witnessing the collapse, incoming of the emergency call, or instance of EMS alerting was considered the start time for CPR. In cases without bystander CPR, we considered initiation of CPR as the instance when the ALS team had arrived on scene. If the ALS team had witnessed the collapse, we considered CPR to be initiated at the time of collapse.

The ROSC after cardiac arrest (RACA) score is an equation for calculating the probability of ROSC, our primary endpoint. The RACA score, which is described elsewhere, accounts for prognostic factors that influence resuscitation success i.e. age, sex, presumed aetiology, location of arrest, bystander CPR, presenting cardiogram rhythm, period until EMS arrival, and whether the collapse has been witnessed.[19] Using the RACA score the expected ROSC rate for both groups was calculated and compared to the observed rate of ROSC for both groups.

#### Statistics

We performed the following statistical tests for significance: Pearson’s chi-squared (χ^2^) test, Kruskal-Wallis test, and Mann-Whitney U test.

Pre-hospital therapeutic measures may differ between mechanical CPR and manual CPR group, therefore as well as calculating the RACA score, further adjustment was required to account for EMS intervention. We performed univariate analysis of epidemiologic factors and EMS treatment. All epidemiological factors included in the RACA score, as well as pre-hospital therapeutic measures with significant influence on ROSC, were included in the multivariate logistic regression analysis. As the duration of CPR was considered a critical variable, we calculated models with and without the duration of CPR.

Predictive analytics were performed using IBM SPSS Statistics Version 22.

According to best scientific practice, p values ≤0.05 are considered to be significant. Unless specified otherwise, continuous data was reported as means and standard deviation (SD), or 95% confidence interval (CI), and frequencies are expressed as percentages.

Ulm University independent ethics board (ref. no. 90/13), and German Resuscitation Registry scientific advisory board approved this study. The data in the German resuscitation registry are fully anonymized. Therefore the consent to participate is not applicable for the analysis. Protocol forms are available on the Internet (www.reanimationsregister.de).[17]

## Results

Mechanical CPR devices (Table 1) were increasingly applied during ALS. S1 Table shows the course over time of the application of the devices recorded in the registry. In 912 cases (4.7%) mechanical CPR devices were used (407 AutoPulse and 505 LUCAS). The control group consists of 18,697 cases of manual-only CPR.

Within the two study groups, population was not distributed uniformly (S2 Table). Age, sex, and presumed aetiology differed significantly between the two groups. The devices were applied more often in public places, while they were used less frequently at home, nursing home, or other medical institutions. Asystole was more frequent in the manual CPR group, and ventricular fibrillation in the mechanical CPR group. The devices were used significantly more frequently when cardiac arrest was witnessed, and bystander CPR was initiated.

More therapeutic measures were performed if mechanical CPR was applied. This observation was significant for defibrillation, intraosseous infusion, tracheal intubation, sodium bicarbonate, epinephrine, and amiodarone application, as well as thrombolysis.

Time interval data (period until arrival of professional help, duration of CPR until ROSC, duration of CPR until death and total duration of the CPR) were not distributed uniformly for the majority of variables: Witnessed arrest, bystander CPR, VF as presenting rhythm, defibrillation, intravenous infusion, tracheal intubation, supraglottic airway, and medication was more frequent with mechanical CPR. The only exception is the mean interval until arrival of professional aid, where values did not differ significantly. The duration of CPR (in total, until first ROSC, and until death) was significantly longer, and patients were significantly younger, and predominantly male, when mechanical CPR devices were applied (S2 Table).

### Factors influencing the choice to apply a mechanical CPR device

**Fig 2:**
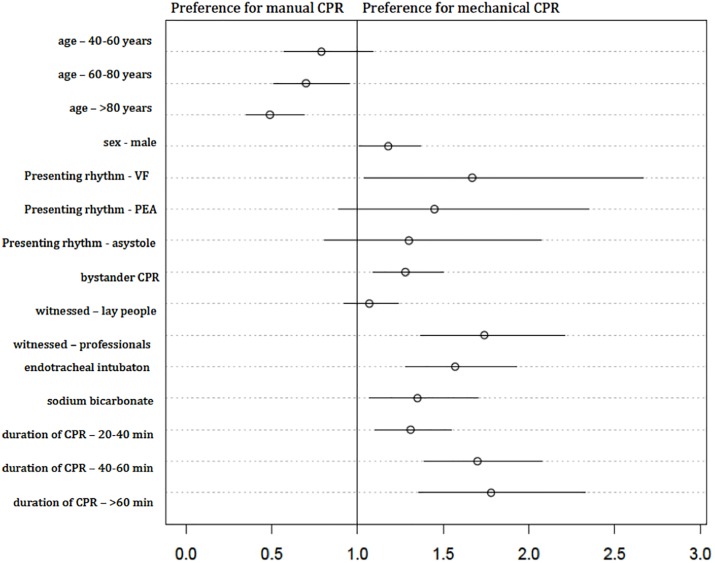
Factors influencing the choice to apply a mechanical CPR device. CPR = cardiopulmonary resuscitation; VF = ventricular fibrillation; PEA = pulseless electric activity.

Using forward selection, we identified factors influencing the decision to apply a mechanical CPR device. A total of seventeen records lacked information, so we included n = 19,592 cases. S3 Table and Fig 2 describes the variables included.

We also found that the older the patients, the higher the probability for receiving manual CPR. Devices were applied predominantly when the incident had been witnessed and when bystander CPR had been performed. A mechanical CPR device was more often applied, when the first witnessed ECG rhythm was ventricular fibrillation. More invasive measures like tracheal intubation, and administration of sodium bicarbonate, had been undertaken in the mechanical CPR group.

Table 2 describes the influence of therapeutic measures on ROSC. The overall percentage of ROSC was 41.2% during the study period. ROSC significantly increased from 38.2% in 2007–2010 to 42.1% in 2011–2014 (S4 Table).

**Table 2:** Valid cases concerning therapeutic measures.

Therapeutic measures	Missing	With treatment		Without treatment		p
		n	ROSC	n	ROSC	
defibrillation	0	8887	4883 (54.9%)	10722	3164 (29.5%)	<0.001
intraosseous infusion	0	1549	588 (38.0%)	18060	7459 (41.3%)	0.01
tracheal intubation	0	15656	7101 (45.4%)	3953	946 (23.9%)	<0.001
supraglottic airway	0	5115	2005 (39.2%)	14494	6042 (41.7%)	0.002
thrombolysis	0	1103	558 (50.6%)	18506	7489 (40.5%)	<0.001
sodium bicarbonate	15	1197	521 (43.5%)	18397	7511 (40.8%)	0.07
epinephrine	0	16589	6901 (41.6%)	3020	1146 (37.9%)	<0.001
amiodarone	17	4846	2914 (60.1%)	14746	5117 (34.7%)	<0.001

ROSC = return of spontaneous circulation.

### Comparison of resuscitation success

**Fig 3:**
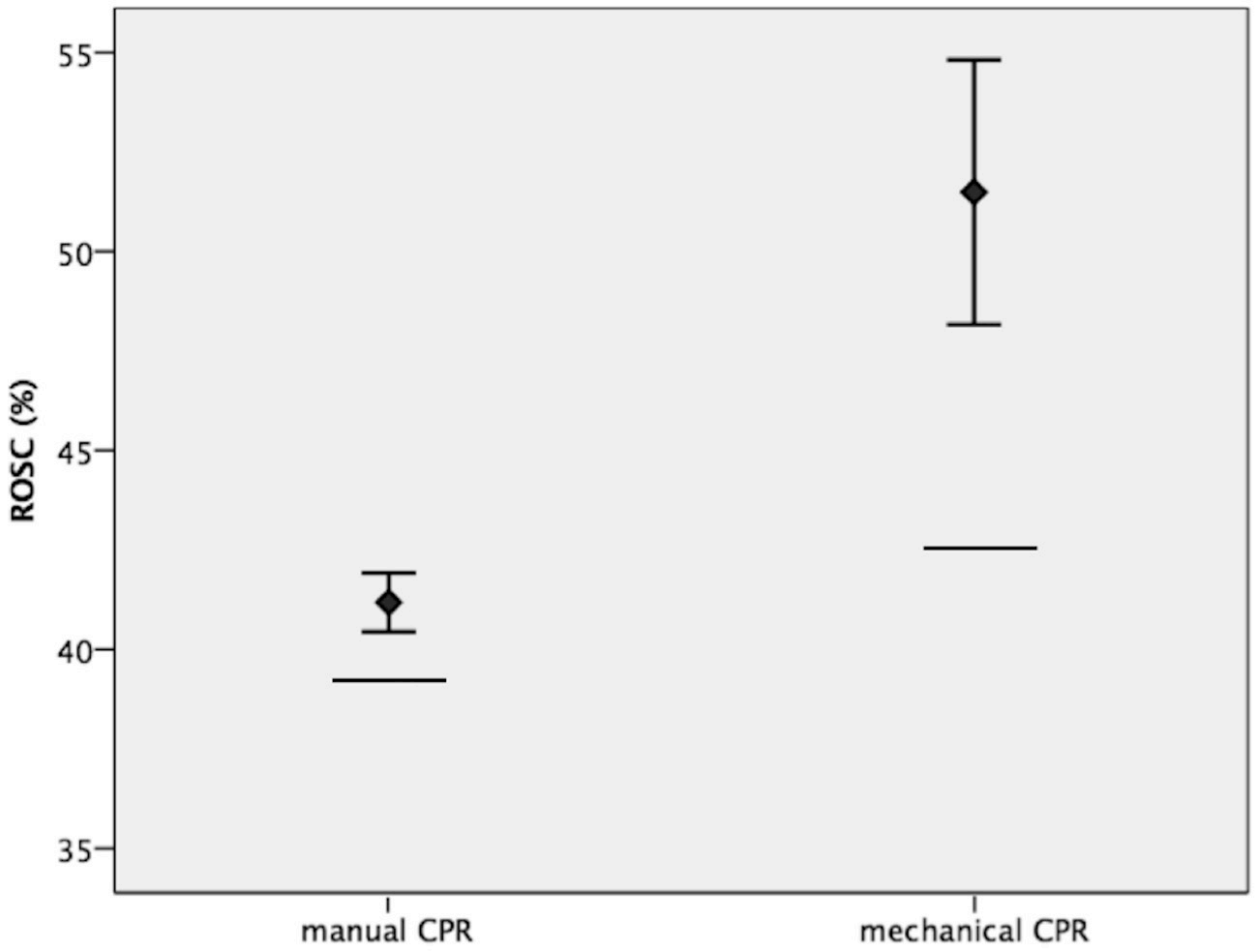
Expected (bar) and observed (diamond; antennas display 95% confidence interval with small bars imaging upper and lower bound) ROSC rates (%). ROSC = return of spontaneous circulation; CPR = cardiopulmonary resuscitation.

Manual CPR resulted in a significantly higher ROSC percentage than expected (41.2%; 95%-CI 40.4–41.9, RACA 39.2%). In the mechanical CPR group, the proportion of ROSC observed was significantly higher (51.5%; 95%-CI 48.2–54.8%) than expected (RACA 42.5%) (Table 3 and Fig 3). For analyses comparing expected to observed rate of ROSC a total of 17,957 patients were included as their RACA score could be calculated, we had to exclude patients whose RACA score was incalculable because of missing variables (n = 1,652).

**Table 3:** Comparison of observed and predicted return of spontaneous circulation (ROSC).

	criteria	manual CPR	mechanical CPR	Pearson’s χ^2^ p
All cases		18697	912	
Excluded (RACA incalculable)		1612	40	
	n	17085	872	
	ROSC (observed)	7036 (41.2%) 95% CI 40.4–41.9%	449 (51.5%) 95% CI 48.2–54.8%	<0.001
	ROSC (expected = RACA score)	39.2%	42.5%	
	ROSC at hospital admission	6717 (39.3%)	420 (48.2%)	<0.001
Witnessed arrest and bystander CPR	n	2755	180	
	ROSC (observed)	1588 (57.6%) 95% CI 55.8–59.5%	117 (65.0%) 95% CI 58.0–72.0%	0.053
	ROSC (expected = RACA score)	52.9	53.2	
	ROSC at hospital admission	1531 (55.6%)	116 (64.4%)	0.020

CPR = cardiopulmonary resuscitation; RACA = ROSC after cardiac arrest; ROSC = return of spontaneous circulation; CI = confidence interval.

### Multivariate logistic regression analysis

After adjustment for epidemiologic factors and EMS treatment but not duration of CPR, odds ratio for ROSC are 1.27 (95%-CI 1.09–1.48) with mechanical vs. manual CPR. After adjustment for duration of CPR the model shows significant benefits for mechanical over manual CPR (OR 1.77 (95%-CI 1.48–2.12) for mechanical CPR) (S5 Table). Comparison of both models confirmed CPR duration as an essential influencing factor for CPR success.

## Discussion

In our study, the use of mechanical CPR devices tend to a ROSC rate that exceeded the expected rate calculated using the RACA score. After adjustment for influencing factors, mechanical CPR remained a significant predictor of ROSC, independent of CPR duration. Considering that resuscitation attempts of less than five minutes had been excluded from our analysis, our data support the hypothesis that the application of the devices may increase survival, especially in particular indications like prolonged CPR.

We consider these findings as important because data about the practical application and benefits from mechanical CPR devices has been limited to date, with many of the conclusions derived from extrapolations of related medical fields or animal experiments. Registries represent the real-life resuscitation environment therefore our analysis constitutes an important addition to clinical and experimental trials.

While our results suggest a benefit of mechanical CPR devices, it is important to emphasise that the use of these devices should be limited to particular circumstances. For example, mechanical CPR devices may not be suitable for use for patients with certain anatomical conditions or traumatic injuries. Trained ALS providers will be able to adjust chest compressions to the individual needs of such patients, whereas a device will not. Another important consideration is that in order to attach a mechanical CPR device properly to a patient, CPR has to be interrupted causing a lower compression fraction.[14, 15] As for any other resuscitation measure the best moment to establish mechanical CPR during the resuscitation process must be considered. Finally, in our study we included only cases where the resuscitation attempt had exceeded five minutes. This is because use of mechanical CPR is not indicated in resuscitation attempts of short duration, as the outcome in this early period is not affected by the CPR mode. As the duration of a resuscitation attempt becomes prolonged, more invasive measures may be used to try and prevent the imminent death of a patient. In such scenarios, an emergency physician’s decision is required if mechanical CPR is indicated or not to avoid over-treatment of patients who have no prospect of survival.

### Context with previous works

With respect to ROSC, previous studies show heterogeneous data, with differing case numbers and differing study designs. The most common outcome variables are haemodynamic parameters and ROSC, or short-term survival.[10, 11] Recently, three major trials have been published. The CIRC trial compared AutoPulse to manual CPR and reported poorer hospital admission and 24-hour survival after AutoPulse CPR, but equal hospital discharge rates and neurology.[14] The LINC trial did not show any significant differences between LUCAS and manual CPR with respect to ROSC, short- or long-term survival, and neurological outcome.[20] The PARAMEDIC trial also failed to show any significant differences in survival, reporting even worse neurological outcome after LUCAS CPR.[21] A recently updated Cochrane Review investigated the application of mechanical CPR devices in six different trials and found that there was insufficient evidence to conclude either benefit or harm from these mechanical devices and advised that more studies are needed.[10] Another systematic review showed significantly higher ROSC with mechanical CPR, especially in the subgroup of the device manufactured by the sponsor of this meta-analysis.[11]

### Limitations

As an analysis of a national resuscitation registry, our study is limited by certain factors, which are common in any registry analysis. Generally, observational studies can demonstrate associations, but not causation. Data quality depends on the participants’ documentation accuracy. We had to exclude 37.3% of the datasets due to missing entries. Participation is on a voluntary basis, and not all German EMS systems contribute to the database. As it is not mandatory, each EMS system may decide on its own whether or not to implement mechanical CPR devices into their ALS algorithm. Little is known about the background of the patients, and their pre-existing conditions, however this is the case for the majority of OHCA studies. Additionally, we do not know the exact circumstances of the resuscitation attempt. For example, we do not have information about interruptions or the no-flow time, complications that may have occurred, or compression depth. As an endpoint, ROSC can easily be obtained, but ROSC is only a surrogate parameter for survival. Unfortunately, in most of the cases follow-up was not reported to the registry by admitting hospitals. Hence, in our study we could not follow long-term outcome parameters like survival and neurological state at hospital discharge.

Nevertheless, registry analyses do have some additional advantages compared with clinical studies. They represent the real-world population and are more representative of everyday performance which is not represented in a controlled clinical trial. Additionally, registries include patients who may not have met the inclusion criteria of prospective randomized clinical trials.

### Conclusion and implications for practice

Mechanical CPR was associated with an increased rate of ROSC, and even when adjusted for risk factors, there is also an indication for advantage over manual CPR. This observation is independent of consideration of CPR duration in multivariate regression analysis. Mechanical CPR devices may increase survival and should be considered in particular indications according to a physicians’ decision, especially during prolonged resuscitation. Further studies are necessary to evaluate the effect of mechanical CPR on short and long-term outcome.

## Supporting information

S1 Table Included cases by CPR mode categorised by year of incident. CPR = cardiopulmonary resuscitation.
(DOCX)

S2 Table Distribution of cases included. VF = ventricular fibrillation; PEA = pulseless electrical activity; CPR = cardiopulmonary resuscitation; EMS = emergency medical service; min = minutes; ROSC = return of spontaneous circulation.
(DOCX)

S3 Table Factors influencing the decisions to apply a mechanical cardiopulmonary resuscitation (CPR) device. See also Fig 2. CI = confidence interval; VF = ventricular fibrillation; PEA = pulseless electrical activity; CPR = cardiopulmonary resuscitation; min = minutes. Not shown in the equation: location of arrest, presumed aetiology, defibrillation and thrombolysis.
(DOCX)

S4 Table Return of spontaneous circulation (ROSC) during the study period. ROSC = return of spontaneous circulation; CoSTR = International Consensus on Cardiopulmonary Resuscitation and Emergency Cardiovascular Care Science with Treatment Recommendations.
(DOCX)

S5 Table Regression model. Odds ratio given with its 95% confidence interval. PEA = pulseless electrical activity; CPR = cardiopulmonary resuscitation; CI = confidence interval; CoSTR = International Consensus on Cardiopulmonary Resuscitation and Emergency Cardiovascular Care Science with Treatment Recommendations.
(DOCX)

S6 Table Dataset definition of minimal underlying data. (XLSX)

S7 Table Minimal underlying dataset. (XLSX)
