# Supplementary material for: Application of mechanical cardiopulmonary resuscitation devices and their value in out-of-hospital cardiac arrest: A retrospective analysis of the German Resuscitation Registry
Source: PLoS One. 2019 Jan 2;14(1):e0208113. doi: 10.1371/journal.pone.0208113 (PMC6314607; doi:10.1371/journal.pone.0208113)
Supplement: S1 Table — CPR = cardiopulmonary resuscitation. (DOCX) [file pone.0208113.s001.docx]

|  | **year** | | | | | | | |
| --- | --- | --- | --- | --- | --- | --- | --- | --- |
|  | **2007** | **2008** | **2009** | **2010** | **2011** | **2012** | **2013** | **2014** |
| all cases | 859 | 1254 | 1649 | 1779 | 2596 | 3271 | 3505 | 4696 |
| ROSC | 309 (36.0%) | 479 (38.2%) | 637 (38.6%) | 693 (39.0%) | 1022 (39.4%) | 1320 (40.4%) | 1580 (45.1%) | 2007 (42.7%) |
|  | | | | | | | | |
| manual CPR | 846 (98.5%) | 1211 (96.6%) | 1555 (94.3%) | 1709 (96.1%) | 2518 (97.0%) | 3169 (96.9%) | 3288 (93.8%) | 4401 (93.7%) |
| mechanical CPR | 13 (1.5%) | 43 (3.4%) | 94 (5.7%) | 70 (3.9%) | 78 (3.0%) | 102 (3.1%) | 217 (6.2%) | 295 (6.3%) |
| -AutoPulse**^®^** | 2 | 37 | 34 | 44 | 31 | 42 | 110 | 107 |
| - LUCAS**^®^** | 11 | 6 | 60 | 26 | 47 | 60 | 107 | 188 |
