# Supplementary material for: Application of mechanical cardiopulmonary resuscitation devices and their value in out-of-hospital cardiac arrest: A retrospective analysis of the German Resuscitation Registry
Source: PLoS One. 2019 Jan 2;14(1):e0208113. doi: 10.1371/journal.pone.0208113 (PMC6314607; doi:10.1371/journal.pone.0208113)
Supplement: S2 Table — VF = ventricular fibrillation; PEA = pulseless electrical activity; CPR = cardiopulmonary resuscitation; EMS = emergency medical service; min = minutes; ROSC = return of spontaneous circulation. (DOCX) [file pone.0208113.s002.docx]

| criteria | CPR mode | | p-value |
| --- | --- | --- | --- |
|  | **manual** | **mechanical** |  |
| n | 18697 | 912 |  |
| age | 69.9 ±14.6 | 66.0 ± 14.8 | <0.001 |
| - valid cases | 18697 | 912 |  |
| sex |  |  |  |
| - male | 12200 (65.3%) | 655 (71.8%) | <0.001 |
| - female | 6437 (34.4%) | 256 (28.1%) |  |
| - not specified | 60 (0.3%) | 1 (0.1%) |  |
| location of arrest |  |  | <0.001 |
| - at home | 12530 (67.0%) | 584 (64.0%) |  |
| - nursing home | 1732 (9.3%) | 50 (5.5%) |  |
| - workplace | 343 (1.8%) | 26 (2.9%) |  |
| - doctor’s office | 297 (1.6%) | 19 (2.1%) |  |
| - public place | 2668 (14.3%) | 169 (18.5%) |  |
| - medical institution | 388 (2.1%) | 12 (1.3%) |  |
| - other place | 613 (3.3%) | 47 (5.2%) |  |
| - not specified | 126 (0.7%) | 5 (0.5%) |  |
| presenting rhythm |  |  | <0.001 |
| - VF | 4650 (24.9%) | 300 (32.9%) |  |
| - PEA | 3048 (16.3%) | 148 (16.2%) |  |
| - asystole | 10467 (56.0%) | 443 (48.6%) |  |
| - others | 305 (1.6%) | 15 (1.6%) |  |
| - not specified | 227 (1.2%) | 6 (0.7%) |  |
| bystander CPR | 4416 (23.6%) | 276 (30.3%) | <0.001 |
| witnessed |  |  | <0.001 |
| - lay people | 9006 (48.2%) | 465 (51.0%) |  |
| - first responder | 344 (1.8%) | 20 (2.2%) |  |
| - ambulance team | 496 (2.7%) | 33 (3.6%) |  |
| - emergency physician | 710 (3.8%) | 49 (5.4%) |  |
| - not witnessed | 8140 (43.5%) | 344 (37.7%) |  |
| - not specified | 1 (0.0%) | 1 (0.1%) |  |
| presumed aetiology |  |  | 0.04 |
| - cardiac | 15036 (80.4%) | 762 (83.6%) |  |
| - hypoxia | 1924 (10.3%) | 79 (8.7%) |  |
| - intoxication | 249 (1.3%) | 16 (1.8%) |  |
| - others | 1488 (8.0%) | 55 (6.0%) |  |
| defibrillation | 8407 (45.0%) | 480 (52.6%) | <0.001 |
| - valid cases | 18697 | 912 |  |
| intraosseous infusion | 1411 (7.5%) | 138 (15.1%) | <0.001 |
| - valid cases | 18697 | 912 |  |
| tracheal intubation | 14856 (79.5%) | 800 (87.7%) | <0.001 |
| - valid cases | 18697 | 912 |  |
| supraglottic airway | 4833 (25.8%) | 282 (30.9%) | 0.001 |
| - valid cases | 18697 | 912 |  |
| thrombolysis | 1018 (5.4%) | 85 (9.3%) | <0.001 |
| - valid cases | 18697 | 912 |  |
| sodium bicarbonate | 1107 (5.9%) | 90 (9.9%) | <0.001 |
| - valid cases | 18684 | 910 |  |
| epinephrine | 15728 (84.1%) | 861 (94.4%) | <0.001 |
| - valid cases | 18697 | 912 |  |
| amiodarone | 4531 (24.3%) | 315 (34.6%) | <0.001 |
| - valid cases | 18682 | 910 |  |
| period until EMS arrival (min) | 9.0 ± 6.3 | 8.9 ± 6.5 | 0.64 |
| - valid cases | 17444 | 882 |  |
| duration CPR until first ROSC (min) | 21.5 ± 13.9 | 26.5 ± 16.9 | <0.001 |
| - valid cases | 7583 | 464 |  |
| duration CPR until death (min) | 33.3 ± 17.7 | 41.1 ± 18.5 | <0.001 |
| - valid cases | 11114 | 448 |  |
| total duration of CPR (min) | 28.5 ± 17.2 | 33.6 ± 19.1 | <0.001 |
| - valid cases | 18697 | 912 |  |
