# Supplementary material for: Application of mechanical cardiopulmonary resuscitation devices and their value in out-of-hospital cardiac arrest: A retrospective analysis of the German Resuscitation Registry
Source: PLoS One. 2019 Jan 2;14(1):e0208113. doi: 10.1371/journal.pone.0208113 (PMC6314607; doi:10.1371/journal.pone.0208113)
Supplement: S3 Table — See also Fig 2. CI = confidence interval; VF = ventricular fibrillation; PEA = pulseless electrical activity; CPR = cardiopulmonary resuscitation; min = minutes. Not shown in the equation: location of arrest, presumed aetiology, defibrillation and thrombolysis. (DOCX) [file pone.0208113.s003.docx]

|  | **p-value** | **odds ratio (95% CI)** |
| --- | --- | --- |
| age – 40-60 years | 0.15 | 0.788 (0.572-1.087) |
| age – 60-80 years | 0.02 | 0.699 (0.513-0.953) |
| age – >80 years | <0.001 | 0.491 (0.350-0.689) |
| sex – male | 0.04 | 1.177 (1.011-1.370) |
| presenting rhythm – VF | 0.03 | 1.667 (1.039-2.674) |
| presenting rhythm – PEA | 0.13 | 1.449 (0.894-2.348) |
| presenting rhythm – asystole | 0.28 | 1.296 (0.812-2.071) |
| bystander CPR | 0.002 | 1.279 (1.095-1.495) |
| witnessed – lay people | 0.39 | 1.069 (0.919-1.244) |
| witnessed – professionals | <0.001 | 1.737 (1.365-2.211) |
| endotracheal intubation | <0.001 | 1.574 (1.283-1.931) |
| sodium bicarbonat | 0.01 | 1.347 (1.067-1.702) |
| duration of CPR – 20-40 min | 0.002 | 1.305 (1.100-1.548) |
| duration of CPR – 40-60 min | <0.001 | 1.699 (1.386-2.082) |
| duration of CPR – >=60 min | <0.001 | 1.782 (1.361-2.334) |
