# Supplementary material for: Application of mechanical cardiopulmonary resuscitation devices and their value in out-of-hospital cardiac arrest: A retrospective analysis of the German Resuscitation Registry
Source: PLoS One. 2019 Jan 2;14(1):e0208113. doi: 10.1371/journal.pone.0208113 (PMC6314607; doi:10.1371/journal.pone.0208113)
Supplement: S4 Table — ROSC = return of spontaneous circulation; CoSTR = International Consensus on Cardiopulmonary Resuscitation and Emergency Cardiovascular Care Science with Treatment Recommendations. (DOCX) [file pone.0208113.s004.docx]

| **period (guidelines)** | **number of cases (n)** | **ROSC** | **Pearson’s χ^2^**  **p** |
| --- | --- | --- | --- |
| 2007-2010 (CoSTR 2005) | 5541 | 2118 (38.2%) | <0.001 |
| 2011-2014 (CoSTR 2010) | 14068 | 5929 (42.1%) |  |
