# Supplementary material for: Application of mechanical cardiopulmonary resuscitation devices and their value in out-of-hospital cardiac arrest: A retrospective analysis of the German Resuscitation Registry
Source: PLoS One. 2019 Jan 2;14(1):e0208113. doi: 10.1371/journal.pone.0208113 (PMC6314607; doi:10.1371/journal.pone.0208113)
Supplement: S5 Table — Odds ratio given with its 95% confidence interval. PEA = pulseless electrical activity; CPR = cardiopulmonary resuscitation; CI = confidence interval; CoSTR = International Consensus on Cardiopulmonary Resuscitation and Emergency Cardiovascular Care Science with Treatment Recommendations. (DOCX) [file pone.0208113.s005.docx]

| **criteria** | **model without CPR duration** | | **model considering CPR duration** | |
| --- | --- | --- | --- | --- |
|  | **p** | **odds ratio (95% CI)** | **p** | **odds ratio (95% CI)** |
| age |  |  |  |  |
| - >80 years | <0.001 | 0.77 (0.71-0.84) | <0.001 | 0.62 (0.57-0.68) |
| sex: male | <0.001 | 0.76 (0.70-0.81) | <0.001 | 0.77 (0.71-0.84) |
| location of arrest |  |  |  |  |
| - nursing home | 0.002 | 0.82 (0.72-0.93) | <0.001 | 0.75 (0.65-0.86) |
| - doctor’s office | <0.001 | 1.90 (1.45-2.48) | <0.001 | 2.17 (1.57-2.98) |
| - public place | <0.001 | 1.68 (1.53-1.86) | <0.001 | 1.55 (1.38-1.73) |
| - medical institution | 0.12 | 1.20 (0.95-1.52) | 0.32 | 1.34 (1.03-1.75) |
| presenting rhythm |  |  |  |  |
| - PEA | <0.001 | 0.46 (0.41-0.52) | <0.001 | 0.60 (0.52-0.69) |
| - asystole | <0.001 | 0.27 (0.25-0.30) | <0.001 | 0.32 (0.29-0.36) |
| bystander CPR | 0.001 | 1.15 (1.06-1.25) | 0.001 | 2.08 (1.89-2.30) |
| witnessed |  |  |  |  |
| - lay people | <0.001 | 2.08 (1.93-2.25) | <0.001 | 2.16 (1.98-2.35) |
| - professionals | <0.001 | 2.46 (2.16-2.79) | <0.001 | 2.78 (2.40-3.24) |
| presumed aetiology |  |  |  |  |
| - hypoxia | <0.001 | 2.49 (2.24-2.78) | <0.001 | 2.27 (2.01-2.56) |
| - intoxication | <0.001 | 1.87 (1.41-2.47) | <0.001 | 1.91 (1.39-2.62) |
| therapeutic measures |  |  |  |  |
| - defibrillation | <0.001 | 1.27 (1.17-1.39) | <0.001 | 1.70 (1.54-1.88) |
| - intraosseous infusion | <0.001 | 0.79 (0.70-0.90) | 0.15 | 1.11 (0.96-1.28) |
| - tracheal intubation | <0.001 | 2.26 (2.06-2.48) | <0.001 | 3.58 (3.23-3.98) |
| - thrombolysis | 0.03 | 0.85 (0.74-0.98) | <0.001 | 4.38 (3.59-5.34) |
| - amiodarone | <0.001 | 1.34 (1.22-1.46) | <0.001 | 2.49 (2.23-2.78) |
| period until professional aid | <0.001 | 0.97 (0.96-0.98) | <0.001 | 0.98 (0.97-0.99) |
| guideline period |  |  |  |  |
| - 2011-2014 (CoSTR 2010) | <0.001 | 1.54 (1.43-1.67) | <0.001 | 1.58 (1.44-1.73) |
| mechanical CPR | 0.003 | 1.27 (1.09-1.48) | <0.001 | 1.77 (1.48-2.12) |
| duration of CPR |  | - | <0.001 | 0.91 (0.91-0.92) |
| constant | <0.001 | 0.44 | <0.001 | 1.80 |
